# The prevalences of impaired fasting glucose and diabetes mellitus in working age men of North China: Anshan Worker Health Survey

**DOI:** 10.1038/srep04835

**Published:** 2014-04-29

**Authors:** Lei Liu, Chuang Zhou, Hang Du, Kai Zhang, Desheng Huang, Jingyang Wu, Anshan Worker Health Survey Group

**Affiliations:** 1 Department of Ophthalmology, The First Affiliated Hospital, China Medical University, Shenyang, 110001, China; 2 Department of Cadre Health Care, Ansteel Group Hospital, Anshan 114000, China; 3 Department of Rehabilitation acupuncture, Anshan Changda Hospital, 114000, China; 4 Department of Out-patient, Ansteel Group Hospital, Anshan 114000, China; 5 Department of Epidemiology, School of Public Health, China Medical University, Shenyang, 110001, China; 6 Department of Endocrinology and Metabolism, Institute of Endocrinology, Liaoning Provincial Key Laboratory of Endocrine Diseases, The First Affiliated Hospital of China Medical University, Shenyang, 110001, China; 7 Department of Health Medical Center, Ansteel Group Hospital, Anshan 114000, China; 8 A comprehensive list of authors and affiliations appear at the end of the paper.

## Abstract

To investigate the prevalence of impaired fasting glucose (IFG) and total diabetes mellitus (DM) including known diabetes and newly diagnosed diabetes in working age men of North China. A cross-section study was conducted at health medical center of Ansteel Group Hospital in Anshan city of China. 37,345 males between 20–60 years of age were recruited in this study. Age-standardized prevalence of IFG and total DM in these working age men were 25.3% and 8.4%, respectively. The prevalence of IFG and total DM increased, as the age progressed. After multinomial logit analysis, age, systolic blood pressure, drinking, smoking, overweight and obesity, total cholesterol, triglycerides, serum creatinine and blood urea nitrogen were independent risk factors for both IFG and DM. The prevalence rate of IFG in Anshan male workers was higher compared with mainland China overall. Diabetes-related education and popularization of DM prevention programs should be actively carried out with age increasing.

With the rapid increasing obesity and lifestyle changing, the number of people diagnosed with diabetes is increasing worldwide^1^. Previous study has reported that there would be more than 366 million individuals with diabetes among adults 20 or more years of age in 2030^2^. It has became into one of the fastest growing public health problems in our world. This problem could be more seriously in developing countries^3^.

As a larger number of population developing country in world, the prevalence of Type 2 diabetes is increasing in China day by day^4^,^5^. Several large national cross-sectional surveys have been conducted to estimate the prevalence for diabetes in mainland China^6^,^7^. It has been estimated that the number of individuals with diabetes had roughly tripled from 20.8 million in 2000 to 92.4 million in 2007^6^,^8^.

As one of components of prediabetes, Impaired Fasting Glucose (IFG) has been paid more attention by researchers. The prevalence of IFG was 10.6% among urban men in 2002^7^ and 15.5% among urban men in 2007^6^. There were more than 430 million workers in China, approximately 80% of them were males (http://www.stats.gov.cn/tjsj/ndsj/2006/indexeh.htm). So the investigation for their health is very important. To our knowledge, there were few english literatures to report the prevalence of IFG in working age population. In order to determine the prevalence of IFG and total DM including known diabetes and newly diagnosed diabetes in working age men, we conducted this health survey in industrial city (Anshan).

## Results

**Figure 1:**
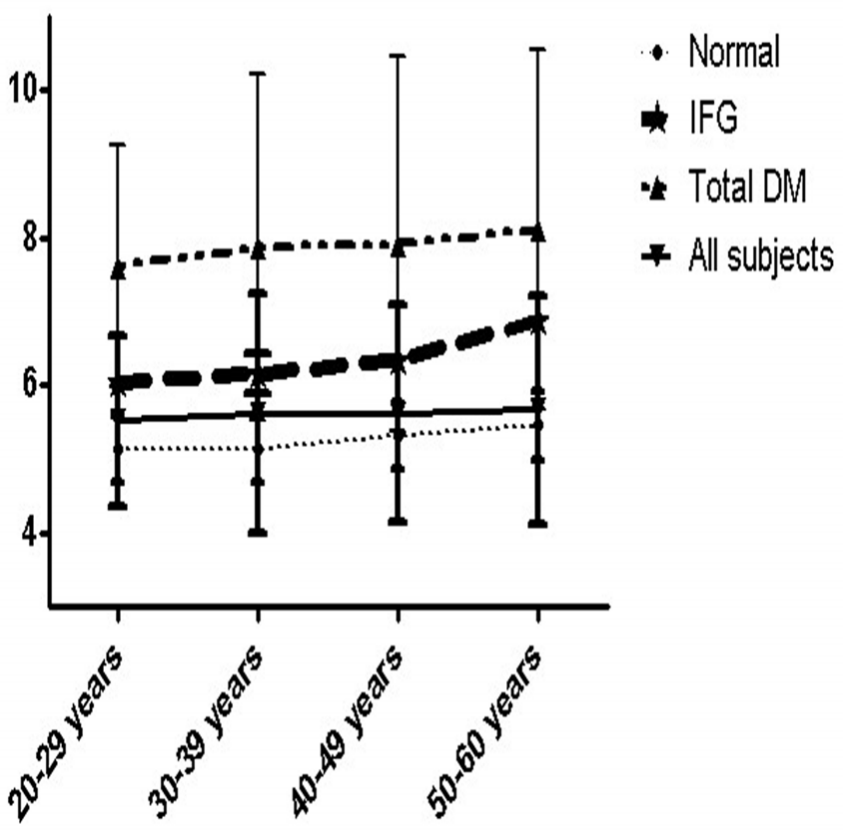
Mean fasting venous plasma glucose concentrations and their standard deviation by age and subject groups in Anshan Worker Health Survey in 2011.

Total of 37,345 over 20 years (20–60 years old, average age 44.69 ± 8.27 yraes) male workers were collected from January to December 2011. Among 37,345 male workers, 47.8% of them were aging form 40–49 years. The sample sizes by age groups were shown in Table 1. The descriptive characteristics of the study samples were presented in Table 2. The pevalence rates of the clinical characteristics in different age groups were shown in Table 3. Age-standardized prevalence of IFG and total DM in these working age men were 25.3% and 8.4%, respectively. In total DM, there were 69.1% newly diagnosed and 30.9% known DM, respectively. Figure 1 showed us that the prevalence of IFG and total DM increased, as the age progressed. In addition, we also found that the fasting glucose levels of total subjects and nomal group increased with the age progressed. In addition, there is no statistically significant for the interaction between age and BMI status in our study (p > 0.05). Multinomial logit models revealed independent risk factors for total DM and IFG (Table 4).

## Discussion

To our knowledge, this was the first research to investigate the prevalence rates of IFG and total DM within male workers in one Chinese industrial city. In order to ensure the quality of study, we used fasting venous plasma glucose test instead of fasting capillary plasma to determine glucose concentrations.

**Figure 2:**
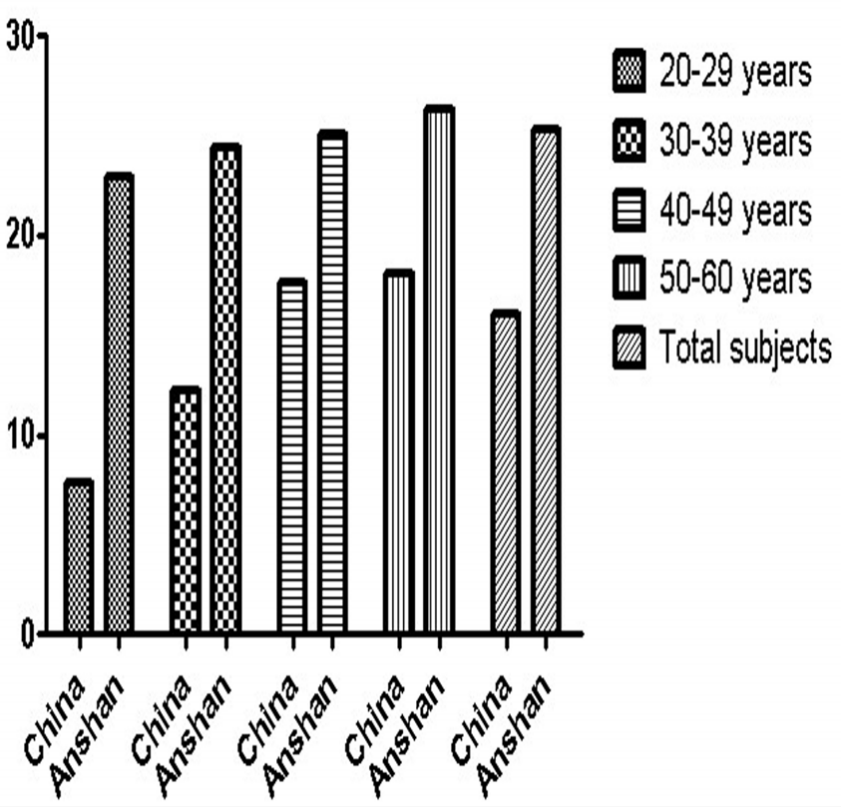
Differences in the prevalence of IFG among urban worker men aged 20–60 years between the 2011 Anshan Worker Health Survey and the 2007 China National Survey^6^.

Our results found that the age-adjusted prevalence of IFG and total DM was 25.3% and 8.4%, respectively. Another study revealed that the overall prevalence of DM was 11.6% in the Chinese adult population and the prevalence among men was 12.1%. The prevalence of newly diagnosed DM was 8.5% in Chinese males^9^. The difference in the prevalence of DM between this study and our investigation might be attributed to different diagnostic criteria. Our research used WHO criteria, whereas Xu et al. used 2010 ADA criteria^9^, which included a glycosylated hemoglobin (HbA_1_c) concentration of 6.5% or higher and 2-hour plasma glucose tests for the diagnosis of DM and might have partly contributed to the increased prevalence of DM. Previous larger simple sizes population-based studies in the past decade has reported the prevalence of IFG and DM was 3.2%–8.23% and 2.7%–9.7%, respectively^6^,^7^,^10^,^11^. During past ten years, there were two national surveys in China^6^,^7^. Among Anshan urban workers aged 20–60 years, the prevalence rate of IFG was significantly higher than the prevalence of IFG reported previously from national representative data^6^ both in different age groups and total subjects (Figure 2). The prevalence of IFG (25.3%) in our investigation was also higher than that (7.1%) in China National Nutrition and Health Survey in 2002^7^. The prevalence of IFG increased with age processed, and it was consistent with the resluts by previous surveys^6^,^7^. Hence, we need to educate the people about precautions to be taken with age increasing and urgent attention to develop a public awareness programme is needded.

**Figure 3:**
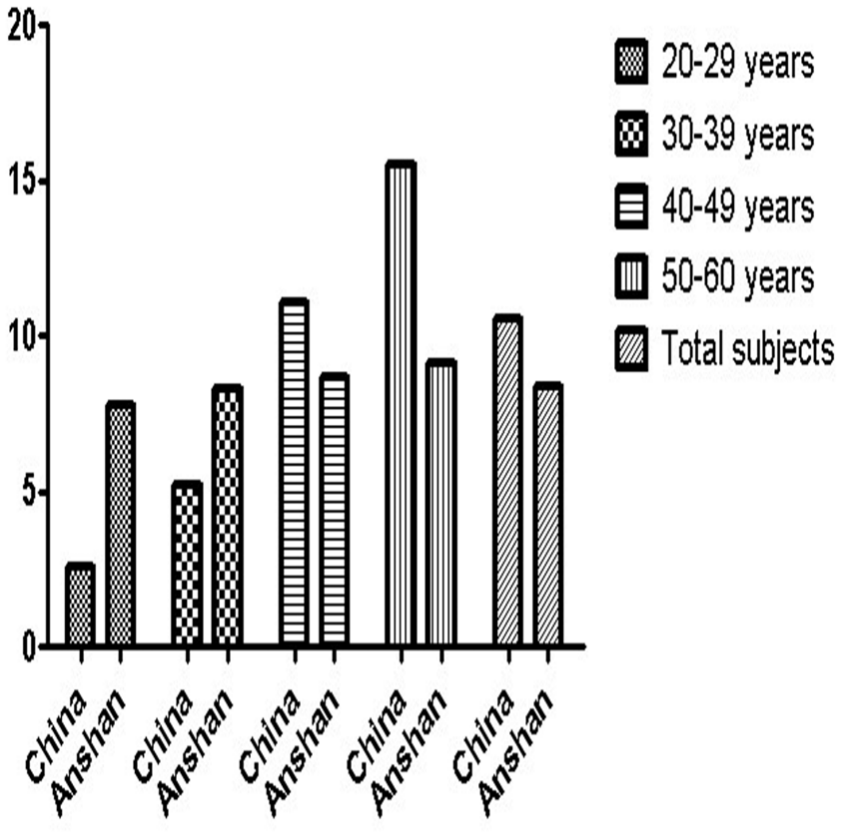
Differences in the prevalence of DM among urban worker men aged 20–60 years between the 2011 Anshan Worker Health Survey and the 2007 China National Survey^6^.

In 20–39 years age group, the prevalence of total DM in Anshan study was higher than that in China national study^6^. However, the results were just the opposite in 40–60 years age group (Figure 3). It could be due to the higher mean BMI (23.5 kg/m^2^) within Anshan study in young aged (20–39 years) than that (23.0 kg/m^2^) in the whole mainland survey^6^. However, the mean BMI (24.0 kg/m^2^) within Anshan study in middle aged (40–60 years) was lower than that (24.3 kg/m^2^) in the survey of mainland China^6^. In all subjects, the prevalence of total DM (8.4%) was lower than that (10.6%) within national survey in 2007, but higher than that (3.9%) in Health Survey in 2002.

Resluts from previous investigations indicated that the prevalence rates of overweight and obesity increased in all age groups and with glucose increasing^12^. But it was inconsistent with our investigation. Because the characteristics of the current study population is different from other studies, there is no significant trends in prevalence of overweight plus obesity and hyperglycemia in Anshan study.

According to different characteristics due to ethnicity, European type 2 DM patients develop the disease at a higher BMI compared with those of East Asian ancestry^13^. The WHO BMI criteria might not be appropriate for the Chinese population, and therefore, we applied Asia-Pacific BMI criteria for overweight and obesity except for WHO BMI criteria. Our results revealed that overweight and obesity were risk factors for IFG and DM according to both the Asia-Pacific and WHO BMI criteria for overweight and obesity. Epidemiological studies have indicated that overweight plus obesity, hypertension, drinking, smoking and hypercholesterolaemia were risk factors for DM even for IFG^14^,^15^,^16^,^17^. It was consistent with our investigation. In addition, our survey also revealed that lower Scr levels was risk factor for both IFG and DM. It may be due to skeletal muscle mass. Serum creatinine is a possible surrogate marker of skeletal muscle mass. Skeletal muscle is one of the major target tissues for insulin, skeletal muscle mass might be associated with hyperglycemia^18^. Surprisingly, our study revealed that higher blood urea nitrogen (BUN) levels was another risk factor for IFG and DM. We reviewed the literatures in Pubmed database, but had not found related reports till now. BUN was considered as a risk factor for diabetes complications such as retinopathy^19^ and cataract^20^.

We found that there was no significant associations of the liver enzymes (Glutamic-Pyruvic transaminase, GPT and Glutamic-Oxalacetic transaminase, GOT levels) with hyperglycemia. However, the study by Schneider et al. suggested that individuals with elevated liver enzymes are at high risk for DM^21^. This controversy needs further research to explain.

Despite the conclusive results that were obtained from this study, there were some shortcomings that should be noted. First, as the reason for limited time of the workers, we did not get a 2 h glucose tolerance test; We could not reveal the true prevalence of DM and impaired glucose tolerance (IGT); Second, because most of workers were men, this study did not investigate female subjects. Third, information on other demographic characteristics such as education level and ethnicity were also collected. Because more than 90% workers were Han ethnicity and 85% of them were primary education levels, we did not analyse these data in this investigation. Fourth, we did not invesigate the type of diabetes in this survey.

In conclusion, the prevalence rate of IFG in Anshan male workers was higher compared within mainland Chinese males^6^. The prevalece of DM was higher in young aged adults but lower in middle aged male workers compared with China national survey^6^. We believe this survey is helpful for better understanding of the prevalence and risk factors for IFG and DM in Chinese working age men, and for early detection and prevention.

## Methods

### Subjects

Anshan is one of the most important industrial cities in North China. At the end of 2010, the population of Anshan city was 3.46 million, of which 1.76 million were men (http://wenwen.soso.com/z/q21324032.htm). A total of 42,680 people were selected and invited to participate in the study; 37,345 working age men which belong to 52 organizations of 12 factories completed the study. The overall response rate was 87.5%. The survey was conducted in Health Medical Center of Ansteel Group Hospital. All health medical workers were intensively trained according to the program of this investigation. According to the fasting glucose levels and self-reports, all the subjects were divided into three groups including normal group, IFG group and total DM group.

### Data collection

Information on age and health status were obtained using a standardized questionnaire. All participants were asked whether they had previously been diagnosed with diabetes. Blood pressure (BP) was measured in the sitting position (first) and supine position (second) at a 5-min interval using an upright standard sphygmomanometer. Height was measured by using a stadiometer. Weight was measured by using a beam balance scale. Body mass index (BMI) was calculated by dividing weight in kilograms by the square of height in meters.

The study was performed in accordance with the principles of the Declaration of Helsinki and was approved by the Ethics Committee of China Medical University. All patients provided written informed consent prior to participating in the study.

### Laboratory methods

Blood was drawn from the antecubital vein for determinations of total cholesterol (TC), high-density lipoprotein cholesterol (HDL-C), low-density lipoprotein cholesterol (LDL-C), triglycerides (TG), fasting plasma glucose levels, serum creatinine (Scr), BUN, GPT and GOT concentrations in the morning after 8 hours fast. All chemistries were measured at a commercially available laboratory.

### Definition of impaired fasting glucose, diabetes mellitus, hypertension and obesity

Diabetes diagnosed according to 1999 WHO criteria^22^. Newly diagnosed DM was defined as a fasting plasma glucose level ≥ 7 mmol/l; Known DM was defined as patients who were diabetics and pursuing diet, exercise and oral hypoglycemic agents and insulin also. IFG was defined as a fasting plasma glucose level from 5.6 to 6.9 mmol/l^23^. Hypertension was defined as a systolic blood pressure (SBP) ⌜ ≥ 140 mmHg or a diastolic blood pressure (DBP) ⌜ ≥ 90 mmHg^24^. Overweight and obesity^25^ were defined as BMI ≥ 25 kg/m^2^ and BMI ≥ 30 kg/m^2^, respectively. In addition, according to Committee of the WHO Western Pacific Region suggestion, the Asia-Pacific BMI criteria^26^ (Overweight and obesity were defined as BMI ≥ 23 kg/m^2^ and BMI ≥ 25 kg/m^2^, respectively.) were applied. Drinking was defined as alcohol intake more than once per month during the past 12 months. Smoking was defined as having smoked 100 cigarettes in one's lifetime and currently smoking cigarettes^9^.

### Statistical analysis

The age-standardized prevalence of IFG and total DM was calculated. Mean ± standard deviation (SD) was used for measurement data. A multinomial logit analysis was applied to estimate the odds ratios (OR) for DM and IFG. Data management and statistical analyses were performed using SPSS statistical software, version 17.0 J (SPSS, Tokyo, Japan) and SUDAAN software, version 10 (Research Triangle Institute). P < 0.05 was considered statistically significant. In multinomial logit analysis, P-values were two-tailed.

## Author Contributions

L.L., C.Z. and D.S.H. have contributed to the design of the study, analysis and interpretation of data and drafting a part of manuscript. J.Y.W., H.D. and K.Z. also took part in analyzing data, and drafting a part of manuscript. A.W.H.S.G. investigated and collected data. L.L., C.Z. and D.S.H. carried out statistical analysis. L.L. and C.Z. prepared all figures and tables. All authors reviewed the manuscript.

## Figures and Tables

**Table 1:** Sample size for fasting glucose by age in this survey

Age in Anshan Worker Health Survey in 2011 (years)	N (%)
20–29	1926(5.2)
30–39	7231(19.4)
40–49	17857(47.8)
50–60	10331(27.6)

**Table 2:** Clinical characteristics of the survey participants in 2011

	Mean (SD)	n (%)
Age, years	44.69 (8.27)	37,345 (100)
Height, cm	172.50 (4.89)	37,345 (100)
Weight, kg	73.66 (10.46)	37,345 (100)
Systolic BP, mmHg	134.52 (18.98)	37,345 (100)
Diastolic BP, mmHg	85.17 (13.49)	37,345 (100)
BMI, kg/m^2^	24.72 (3.10)	37,345 (100)
Total cholesterol, mmol/l	4.92 (0.94)	37,345 (100)
LDL-C, mmol/l	2.76 (0.72)	37,345 (100)
HDL-C, mmol/l	1.33 (0.31)	37,345 (100)
Triglycerides, mmol/l	1.96 (1.84)	37,345 (100)
Fasting glucose, mmol/l	5.62 (1.49)	37,345 (100)
WBC, *10^∧^9/l	6.42 (1.74)	37,345 (100)
GPT, u/l	28.76 (23.16)	37,345 (100)
GOT, u/l	24.66 (14.14)	37,345 (100)
Scr, μmol/l	70.47 (15.88)	37,345 (100)
BUN, μmol/l	5.42 (1.36)	37,345 (100)
Smoking	N/A	27,953 (74.9)
Drinking	N/A	28,476 (76.3)

Abbreviation: total cholesterol, TC; high-density lipoprotein cholesterol, HDL-C; low-density lipoprotein cholesterol, LDL-C; triglycerides, TG; serum creatinine, Scr; blood urea nitrogen, BUN; Glutamic-Pyruvic transaminase, GPT; Glutamic-Oxalacetic transaminase, GOT; body mass index, BMI; white blood cell, WBC; blood pressure, BP; standard deviation, SD; Not Applicable, N/A.

**Table 3:** Prevalence of the clinical characteristics in different age geoups (%)

	20–29 years	30–39 years	40–49 years	50–60 years	≥20 years
Based on fasting plasma glucose					
Normal < 5.5 mmol/l	69.3	67.3	66.2	64.6	66.3
Impaired fasting plasma glucose 5.5–6.9 mmol/l	22.9	24.4	25.1	26.3	25.3
Hyperglycaemia ≥ 7 mmol/l	7.8	8.3	8.7	9.1	8.4
BMI < 25 kg/m^2^	55.8	55.2	55.8	56.1	55.8
BMI ≥ 25 kg/m^2^	44.2	44.8	44.2	43.9	44.2
Systolic BP < 120 mmHg	21.9	21.7	21.3	21.6	21.5
Systolic BP 120–139 mmHg	47.8	46.8	44.6	42.3	44.5
Systolic BP ≥ 140 mmHg	30.3	31.5	34.1	36.1	34
Diastolic BP < 80 mmHg	38.7	36	34.9	34.9	35.3
Diastolic BP 80–89 mmHg	30.8	32.8	32.2	30.8	31.9
Diastolic BP ≥ 90 mmHg	30.5	31.1	32.9	34.3	32.8
TC < 6.2 mmol/l	91.8	92.1	91.3	91.3	91.9
TC ≥ 6.2 mmol/l	8.2	7.9	8.7	8.7	8.1
TG < 2.24 mmol/l	75.7	74.8	74.9	74.6	74.9
TG ≥ 2.24 mmol/l	24.3	25.2	25.1	25.4	25.1
LDL-C < 4.1 mmol/l	96.6	96.5	96.2	96.2	96.3
LDL-C ≥ 4.1 mmol/l	3.4	3.5	3.8	3.8	3.7
HDL-C < 1 mmol/l	10.7	10.9	9.4	9.5	9.7
HDL-C ≥ 1 mmol/l	89.3	89.1	90.6	90.5	90.3
GPT < 40 u/l	82.2	83.3	83.5	84.7	83.2
GPT ≥ 40 u/l	17.5	16.7	16.5	15.3	16.8
GOT < 40 u/l	94	94.9	94.6	94.7	94.5
GOT ≥ 40 ul/l	6	5.1	5.4	5.3	5.5
WBC < 4*10^∧^9/l	4.2	4.5	4.6	4.5	4.4
WBC 4–10*10^∧^9/l	93.5	92.4	92.1	92.3	92.5
WBC > 10*10^∧^9/l	2.3	3.1	3.3	3.2	3.1
Scr < 44 μmol/l	0.5	0.3	0.4	0.4	0.4
Scr 44–133 μmol/l	99.3	99.6	99.4	99.5	99.4
Scr > 133 μmol/l	0.2	0.1	0.2	0.1	0.2
BUN < 3.2 μmol/l	2.2	1.8	1.8	2.3	1.9
BUN 3.2–6 μmol/l	73.6	70.4	68.7	70.2	69.7
BUN > 6 μmol/l	24.2	27.9	29.5	27.5	28.4
Smoking	68.7	75.2	74.8	76.1	74.9
Drinking	75.1	77.2	76.1	76.9	76.3

Abbreviation: total cholesterol, TC; high-density lipoprotein cholesterol, HDL-C; low-density lipoprotein cholesterol, LDL-C; triglycerides, TG; serum creatinine, Scr; blood urea nitrogen, BUN; Glutamic-Pyruvic transaminase, GPT; Glutamic-Oxalacetic transaminase, GOT; body mass index, BMI; white blood cell, WBC; blood pressure, BP.

**Table 4:** Multinomial logit models: Odds Ratios for total DM and IFG

	IFG			Diabetes		
	OR	95%CI	P	OR	95%CI	P
Age, per 10 years increment	2.37	2.01–2.89	<0.01	2.68	2.60–2.77	<0.01
Systolic BP, per increase of 10 mmHg	1.34	1.01–1.98	<0.01	1.39	1.11–1.89	0.01
Diastolic BP, per increase of 10 mmHg	1.09	0.67–2.11	0.11	1.12	0.99–1.51	0.06
Overweight and obesity	1.78	1.41–2.24	<0.01	1.83	1.31–2.44	0.03
Overweight and obesity*	1.91	1.52–2.43	<0.01	1.93	1.61–2.51	<0.01
TC, per increase of 0.56 mmol/l	1.42	1.01–1.78	<0.01	1.47	1.21–1.65	<0.01
LDL-C, per increase of 0.56 mmol/l	1.23	0.87–1.51	0.98	1.27	0.98–1.41	0.65
HDL-C, per increase of 0.56 mmol/l	0.62	0.31–1.02	0.13	0.76	0.44–1.12	0.07
TG, per increase of 0.56 mmol/l	1.04	1.01–1.44	<0.01	1.05	0.98–1.24	0.02
GPT, per increase of 10 u/l	0.99	0.86–1.12	0.06	1.03	0.79–1.33	0.06
GOT, per increase of 10 u/l	1.02	0.88–1.45	0.14	1.04	0.98–1.15	0.06
Scr, per decrease of 0.56 mmol/l	1.33	1.05–1.77	<0.01	1.45	1.15–1.69	0.03
BUN, per increase of 0.56 mmol/l	1.41	1.12–1.79	<0.01	1.77	1.01–2.31	<0.01
Height, per increase of 10 cm	0.89	0.76–1.01	0.08	0.86	0.65–1.05	0.11
Weight, per increase of 10 kg	1.01	0.86–1.21	0.06	1.12	0.78–1.41	0.12
WBC, per increase of 1*10^∧^9/l	0.87	0.65–1.11	0.10	0.76	0.54–1.04	0.15
Smoking	1.12	0.99–1.31	0.06	1.23	1.01–1.42	0.03
Drinking	1.33	1.04–1.63	0.02	1.35	1.11–1.59	0.01

Abbreviation: total cholesterol, TC; high-density lipoprotein cholesterol, HDL-C; low-density lipoprotein cholesterol, LDL-C; triglycerides, TG; serum creatinine, Scr; blood urea nitrogen, BUN; Glutamic-Pyruvic transaminase, GPT; Glutamic-Oxalacetic transaminase, GOT; blood pressure, BP; odds ratios, OR; impaired fasting glucose, IFG; confidence intervals, CI; diabetes mellitus, DM; white blood cell, WBC. Overweight and obesity* were defined as BMI ≥ 23 kg/m^2^ and BMI ≥ 25 kg/m^2^, respectively.

## References

[b1] Wild S., Roglic G., Green A., Sicree R. & King H. Global prevalence of diabetes: estimates for the year 2000 and projections for 2030. Diabetes Care 27, 1047–1053 (2004). 15111519 10.2337/diacare.27.5.1047

[b2] Kengne A. P., Amoah A. G. & Mbanya J. C. Cardiovascular complications of diabetes mellitus in sub-Saharan Africa. Circulation 112, 3592–3601 (2005). 16330701 10.1161/CIRCULATIONAHA.105.544312

[b3] Muninarayana C., Balachandra G., Hiremath S. G., Iyengar K. & Anil N. S. Prevalence and awareness regarding diabetes mellitus in rural Tamaka, Kolar. Int J Diabetes Dev Ctries 30, 18–21 (2010). 20431801 10.4103/0973-3930.60005 PMC2859279

[b4] Gao W. G. *et al.* Increasing trend in the prevalence of Type 2 diabetes and pre-diabetes in the Chinese rural and urban population in Qingdao, China. Diabet Med 26, 1220–1227 (2009). 20002473 10.1111/j.1464-5491.2009.02832.x

[b5] Han Z. G., Liu C. X., Pan J., Zhao L. J. & Wang J. L. Prevalence of diabetes mellitus and impaired fasting glucose of health check-up in a sanatorium of Shanghai in 2003 and 2010. Zhonghua Yu Fang Yi Xue Za Zhi 45, 1099–1102 (2011). 22336345

[b6] Yang W. *et al.* Prevalence of diabetes among men and women in China. N Engl J Med 362, 1090–1101 (2010). 20335585 10.1056/NEJMoa0908292

[b7] Liu S. *et al.* Prevalence of diabetes and impaired fasting glucose in Chinese adults, China National Nutrition and Health Survey, 2002. Prev Chronic Dis 8, A13 (2011). 21159225 PMC3044024

[b8] Steinman R. A. & Birshtein B. K. Treatment and awareness of type 2 diabetes in Beijing, China, compared to New York. Diabetes Educ 33, 282–290 (2007). 17426303 10.1177/0145721707299262

[b9] Xu Y. *et al.* Prevalence and control of diabetes in Chinese adults. JAMA 310, 948–959 (2013). 24002281 10.1001/jama.2013.168118

[b10] Wang H. *et al.* Prevalence and determinants of diabetes and impaired fasting glucose among urban community-dwelling adults in Guangzhou, China. Diabetes Metab 35, 378–384 (2009). 19665414 10.1016/j.diabet.2009.03.006

[b11] Gu D. *et al.* Prevalence of diabetes and impaired fasting glucose in the Chinese adult population: International Collaborative Study of Cardiovascular Disease in Asia (InterASIA). Diabetologia 46,1190–1198 (2003). 12879248 10.1007/s00125-003-1167-8

[b12] Wang Y., Mi J., Shan X. Y., Wang Q. J. & Ge K. Y. Is China facing an obesity epidemic and the consequences? The trends in obesity and chronic disease in China. Int J Obes (Lond) 31, 177–188 (2007). 16652128 10.1038/sj.ijo.0803354

[b13] Ma R. C. & Chan J. C. Type 2 diabetes in East Asians: similarities and differences with populations in Europe and the United States. Ann N Y Acad Sci 1281, 64–91 (2013). 23551121 10.1111/nyas.12098 PMC3708105

[b14] Khambalia A. *et al.* Prevalence and risk factors of diabetes and impaired fasting glucose in Nauru. BMC Public Health 11, 719 (2011). 21943388 10.1186/1471-2458-11-719 PMC3187757

[b15] Basit A. *et al.* Temporal changes in the prevalence of diabetes, impaired fasting glucose and its associated risk factors in the rural area of Baluchistan. Diabetes Res Clin Pract 94, 456–462 (2011). 21890227 10.1016/j.diabres.2011.08.009

[b16] Rodríguez-Moran M. *et al.* Obesity and family history of diabetes as risk factors of impaired fasting glucose: implications for the early detection of prediabetes. Pediatr Diabetes 11, 331–336 (2010). 19895410 10.1111/j.1399-5448.2009.00590.x

[b17] Choi B. C. & Shi F. Risk factors for diabetes mellitus by age and sex: results of the National Population Health Survey. Diabetologia 44, 1221–1231 (2001). 11692170 10.1007/s001250100648

[b18] Harita N. *et al.* Lower serum creatinine is a new risk factor of type 2 diabetes: the Kansai healthcare study. Diabetes Care 32, 424–426 (2009). 19074997 10.2337/dc08-1265 PMC2646021

[b19] Zhong Z. L., Han M. & Chen S. Risk factors associated with retinal neovascularization of diabetic retinopathy in type 2 diabetes mellitus. Int J Ophthalmol 4, 182–185 (2011). 22553638 10.3980/j.issn.2222-3959.2011.02.15 PMC3340710

[b20] Xia X., Zhang X. & Xia H. A study of factors related to the incidence of cataract in patients with non-insulin dependent diabetes mellitus. Yan Ke Xue Bao 17, 180–182 (2001). 12567748

[b21] Schneider A. L. *et al.* Liver enzymes, race, gender and diabetes risk: the Atherosclerosis Risk in Communities (ARIC) Study. Diabet Med 30, 926–933 (2013). 23510198 10.1111/dme.12187 PMC3715563

[b22] Puavilai G., Chanprasertyotin S. & Sriphrapradaeng A. Diagnostic criteria for diabetes mellitus and other categories of glucose intolerance: 1997 criteria by the Expert Committee on the Diagnosis and Classification of Diabetes Mellitus (ADA), 1998 WHO consultation criteria, and 1985 WHO criteria. World Health Organization. Diabetes Res Clin Pract 44, 21–26 (1999). 10414936 10.1016/s0168-8227(99)00008-x

[b23] American Diabetes Association. Diagnosis and classification of diabetes mellitus. Diabetes Care 33, S62–S69 (2010). 20042775 10.2337/dc10-S062 PMC2797383

[b24] Stergiou G. S., Karpettas N., Atkins N. & O'Brien E. Impact of applying the more stringent validation criteria of the revised European Society of Hypertension International Protocol 2010 on earlier validation studies. Blood Press Monit 16, 67–73 (2011). 21258237 10.1097/MBP.0b013e32834331e7

[b25] Anuurad E. *et al.* The new BMI criteria for asians by the regional office for the western pacific region of WHO are suitable for screening of overweight to prevent metabolic syndrome in elder Japanese workers. J Occup Health 45, 335–343 (2003). 14676412 10.1539/joh.45.335

[b26] Chang C. J. *et al.* Low body mass index but high percent body fat in Taiwanese subjects: implications of obesity cutoffs. Int J Obes Relat Metab Disord 27, 253–259 (2003). 12587007 10.1038/sj.ijo.802197

